# Patients' perceptions of the role of community pharmacists in the prevention and control of cardiovascular diseases: a qualitative investigation

**DOI:** 10.4314/ahs.v26i2.11

**Published:** 2026-06

**Authors:** Nthabiseng F Motlohi, Kofi B Mensah, Neelaveni Padayachee, Ruwayda Petrus, Saleh F Alqifari, Varsha Bangalee

**Affiliations:** 1 Discipline of Pharmaceutical Sciences, University of KwaZulu-Natal, Durban, South Africa; 2 Faculty of Pharmacy and Pharmaceutical Sciences, Kwame Nkrumah University of Science and Technology, Kumasi, Ghana; 3 Department of Pharmacy and Pharmacology, University of Witwatersrand, Johannesburg, South Africa; 4 Discipline of Psychology, University of KwaZulu-Natal, Durban, South Africa; 5 Department of Pharmacy Practice, University of Tabuk, Tabuk, Saudi Arabia; 6 Department of Health Information Management, Botho University, Maseru, Lesotho

**Keywords:** Cardiovascular diseases, role, community pharmacist, patients' perceptions, prevention and control, qualitative methods

## Abstract

**Background:**

Cardiovascular diseases are a leading cause of mortality globally. Community pharmacists can improve the impact of cardiovascular diseases by early identification of risk factors and provision of health promotion. However, patients' perceptions of community pharmacists are not well understood.

**Objective:**

To understand how patients perceive the involvement of community pharmacists in preventing and controlling cardiovascular diseases.

**Methods:**

Semi-structured interviews were conducted with 10 (n = 10) cardiovascular disease patients who utilise community pharmacy services in Maseru and Berea districts. Convenience sampling method was used to select participants. The questionnaire comprised questions to uncover patients' experiences, utilisation, and expectations of community pharmacists' services. Thematic analysis was employed.

**Results:**

Four themes were identified, namely 1) perceived role of a community pharmacist, 2) patient healthcare-seeking behaviour, 3) perceived community pharmacist care outcomes, and 4) future expectations. Generally, cardiovascular disease patients visited community pharmacists every month for medication refills and were less aware of other services. Overall, patients were appreciative of and were willing to support future community pharmacists' services. The patients expect community pharmacists to lead initiatives that educate people about cardiovascular diseases and standardise services across all pharmacy outlets.

**Conclusions:**

Patients had limited awareness of other services provided by community pharmacists beyond dispensing medication, such as disease detection and health promotion. However, they valued the services provided by community pharmacists and were willing to utilise expanded services. This presents an opportunity for community pharmacists to raise awareness of their current scope of work and expansion of care.

## Background

The role of community pharmacists is evolving globally. The International Pharmaceutical Federation (2020) classifies essential community pharmacy care into four categories: reviewing, dispensing, prescribing, and administering medication^1^. In addition to the core functions, previous reviews have reported effective community pharmacists' health promotion in preventing and controlling CVDs^2^-^4^. Favourable clinical outcomes were reported for diabetes, blood pressure and smoking following community pharmacist interventions that targeted CVD risk factors^3^, ^5^. Similarly, community pharmacists' patient education and medication review interventions improved blood pressure management in patients with CVD risk factors^2^, ^4^, ^5^. These findings highlight the crucial role of community pharmacists in preventing and controlling CVDs. In Lesotho, community pharmacies are recognised as an essential part of the private-for-profit healthcare system that provides care and medicines^6^. However, patients' perceptions of community pharmacists' care need to be better understood. The primary goal of the study was to delve into the experiences and views of CVD patients concerning the role of community pharmacists in preventing and managing CVDs. Specifically, the study sought to answer the following research questions:

How do CVD patients perceive the role of community pharmacists in preventing and controlling CVDs?
What are the experiences of CVD patients with community pharmacists in Lesotho?
What are CVD patients perceived benefits and barriers to utilising community pharmacists' services in preventing and controlling CVDs in Lesotho?
What are the expectations of CVD patients regarding community pharmacists' services in Lesotho?

For the current study, CVD patients are patients with reported CVD risk factors such as hypertension, diabetes, and dyslipidaemia and patients with established CVDs such as stroke.

## Methods

The methodological reporting of this study is guided by the consolidated criteria for reporting qualitative studies^7^. The participants were recruited with the assistance of community pharmacists who had participated in the first leg of a larger mixed methods study. The pharmacists invited the patients and shared their contact details with the researchers. Another invitation for participation was posted in the WhatsApp group for the Lesotho Diabetes Association through the assistance of its founding members, and interested participants contacted the researcher. The researcher contacted the participants via telephone, informed them about the study's objectives, and invited them to participate. Upon acceptance of the invitation, a one-on-one interview was scheduled, and the interview mode (virtual or contact) was agreed upon based on the participants' preferences. Each participant gave a verbal informed consent at the beginning of the interview which was audio-recorded. Participants were included in the study based on the following criteria: aged 18 years or above, have at least one CVD risk factor or an established CVD, be a user of community pharmacy services in Lesotho, and be able to communicate in either English or Sesotho, the native language. Exclusion criteria included patients who were health professionals, current or former community pharmacy employees, or those unwilling to participate in the study. Data was collected from May through July 2023. A semi-structured interview schedule written in English with Sesotho translations was developed based on the literature and modified to meet the study's objectives^8^. Virtual interviews started with general questions such as “How has the experience of living with the disease been?” The core discussion sought to uncover the understanding of community pharmacists' roles, experiences, and expectations of CVD patients towards community pharmacists' services. The interview schedule was pilot-tested with three participants, and modifications were made to the questions for improved clarity. NFM, the first author, conducted the virtual interviews through phone calls. NFM is a Lesotho citizen and speaks both Sesotho and English. The interviews were audio-recorded using a cell phone, the Microsoft 365 dictation tool, and a laptop through the Zoom application^9^,^10^. The interviews were conducted until there were no new emerging themes^11^. Data analysis adopted an iterative process by analysing data while collecting it. NFM transcribed the audio interviews verbatim and translated the transcrips to English. Data analysis followed a 6-step thematic analysis which has 6 steps as outlined by Virginia Braun^12^. The NFM coded the variables, and RP and VB verified the codes against the transcripts. Similar codes were organised and re-arranged into themes and sub-themes. All the authors (NFM, RP, VB, KBM, NP and SFA) verified and agreed upon the final themes and sub-themes. The first author (NFM) is a doctoral student and a trained pharmacist. The co-authors hold doctoral qualifications in pharmacy and psychology. They have broad experiences conducting qualitative research in pharmacy practice, psychology, and other health disciplines.

## Ethical considerations

The study protocol was approved by the Humanities and Social Sciences Research Ethics Committee (HSSREC) of the University of KwaZulu-Natal (Ref: HSSREC/00003205/2021) and the Research and Ethics Committee of the Ministry of Health of Lesotho (Ref: ID101-2021-Renew 01). The interviewer (NFM) contacted the participants through telephone to provide them with an overview of the study. A verbal consent to audio-record the interview was obtained at the start of the interview. Information related to the confidentiality of the interview recordings and the participant's rights were read out to the participants at the beginning of the interview, and further questions were invited to provide clarity. Every participant was given a code, which was used as a patient's identity on the transcripts to conceal their identities. Participation in the study was voluntary and depended on the willingness of the participants to partake in the study.

## Results

A total of 14 participants (n = 14) were interviewed, three (n = 3) of whom were interviewed for the pilot study. The results of the pilot were excluded from the final analysis following modification of the questionnaire while one participant was excluded due to her previous employment at a community pharmacy. The remaining ten respondents (n = 10) were included in the final analysis. On average, the interviews lasted for 30 minutes. The sociodemographic details of the respondents are presented in Table 1.

**Table 1:** Sociodemographic details of the respondents

Respondent	Gender	Age (years)	Employment status	Education level	Self-reported CVDs	How long have you been diagnosed?	Had a stroke/heart attack before?	District
MAS004	Female	60	Retired	Tertiary	Hypertension	14 years	No	Berea
					Diabetes			
MAS005	Female	41	Unemployed	Secondary	Hypertension	7 years	Yes	Maseru
					Diabetes			
MAS006	Female	44	Employed	Tertiary	Hypertension	6 years	No	Berea
					Diabetes			
					Hyperlipidemia			
MAS008	Male	50	Employed	Tertiary	Hypertension	7 years	No	Maseru
					Diabetes			
MAS009	Male	65	Employed	Tertiary	Hypertension	10 years	No	Maseru
					Diabetes			
					Hyperlipidemia			
MAS010	Male	57	Employed	Tertiary	Hypertension	15 years	No	Maseru
					Diabetes			
MAS011	Male	69	Retired	Tertiary	Hypertension	Over 30	Yes	Maseru
					Hyperlipidemia	years		
MAS012	Male	51	Self-employed	Tertiary	Diabetes	1 year	No	Maseru
MAS013	Male	57	Employed	Tertiary	Hypertension	15 years	No	Maseru
					Diabetes			
MAS014	Female	52	Employed	Tertiary	Hypertension	18 years	No	Maseru

**Figure 1:**
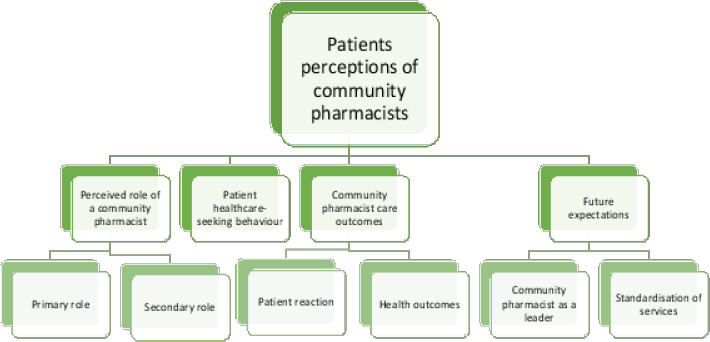
Themes and sub-themes

Four themes with their corresponding subthemes were identified. Figure 1 illustrates the identified themes and sub-themes.

### Perceived role of a community pharmacist

#### Primary role

The respondents described a community pharmacist as an expert and provider of medicine. Their reason for visiting a community pharmacy was to get their prescription refill (Table 2, 2.01 – 2.04). Some get medication from the community pharmacies because of stock out at the clinics, while others are advised by their doctors, indicating an established relationship between doctors and community pharmacists (Table 2, 2.01 – 2.04). Furthermore, community pharmacists respond to individual medicine requirements by placing medication orders to meet individual prescriptions and calling patients to check and remind them about prescription refill (Table 2, 2.03, 2.05). The respondents acknowledged community pharmacists as drug experts due to their ability to suggest alternatives when the prescribed brand is unavailable (Table 2, 2.02).

**Table 2:** Respondents' excerpts

Excerpt no.	Excerpts
**2.01**	*“When my medication runs out every month, and I do not go for a check-up for the doctor to give me medicine, I go to the chemist to buy it.” MAS014.*
**2.02**	*“I go there looking for medicines as the prescription is already there. I go there and tell them what I need, and they give me the medicines. If the one I want is not available, they can suggest another alternative.” MAS012.*
**2.03**	*“I know that at the pharmacy, they will try by all means to make sure they order this medication and have it in stock, ready for my collection…it was my doctor who advised me about them…if I do not go on the scheduled date, they would call to remind me that I am supposed to come and refill your medication.” MAS011.*
**2.04**	*“I collect my medication from [hospital name]. Sometimes they disperse less than prescribed, then I will collect the rest at the pharmacy.” MAS004.*
**2.05**	*“I work in remote areas… After a while, they will want to know if I am still well and if the medication is still there. They call me.” MAS010.*
**2.06**	*“When I feel like my sugar is low or high, I go to a chemist to get tested.” MAS005.*
**2.07**	*“Usually, when I want to do tests, I see a doctor. I know there are other services, but I have never sought them.” MAS010.*
**2.08**	*“Besides the prescription refill, there is nothing else that I know of.” MAS013*
**2.09**	*“I have got a blood sugar machine at home. They recommended that I should have my machine at home.” MAS006.*
**2.10**	*“…I am always encouraged to exercise. …They advise against eating foods that increase blood sugar and blood pressure and saturated foods like that…Avoid sugar and fatty meals. Consume vegetables.” MAS006.*
**2.11**	*“Sometimes they do other tests like taking your weight and height so that eventually you know what your BMI is, …and therefore that is what they do.” MAS010.*
**2.12**	*“…other things like my weight and my height that will eventually establish my BMI that is how they help me. If I see that my BMI is above normal, it is just communication that they encourage me to make sure that I exercise. I must do all sorts of things related to that.” MAS009.*
**2.13**	*“At the pharmacy, they will perform tests and advise me to come back tomorrow to check if the readings have gone down. If it does not go down, they would refer you to a doctor.” MAS004.*
**2.14**	*“…community pharmacists are closer to the people. So again, the queues are not there…I prefer the one in town because the hypertension medication is cheaper and affordable. The one close to home, they have never said medicine has run out and come back on Monday. Even with the one in town, I find the medicines available when I arrive.” MAS008.*
**2.15**	*“There are many of them even here in … [name of a residential place], but I never use them. I am not comfortable buying them just anywhere. So, I only use one pharmacy in town. When you walk into a pharmacy, you check how clean it is. You look at the staff and their approach or conduct when they assist you. It makes you want to go back to get services again. Where I go, they are very good… But in most cases, I use only the medical aid, and I am fine” MAS014.*
**2.16**	*“…and find someone with a bright face, who knows my name, with whom we work on a personal level…he respects me…He works with me according to my health needs…I feel satisfied.” MAS004.*
**2.17**	*“I use certain pharmacies. I believe there are two that I take medication from. One is near me when I am at work and is in town. the other is near me when I am at home.*” *MAS010.*
**2.18**	*“Cash. I have a medical aid, but I do not use it to buy medicines. Looking at the price, I would not use it.” MAS012.*
**2.19**	*“I get insulin from the government clinic and go to a pharmacy at least once a month, mostly for blood pressure medication.” MAS005.*
**2.20**	*“You know I can pay for them because they care for us. At the pharmacy, upon arrival, they examine the medical booklet…talk to you first, and ask questions. Even if you come and tell them, there is a medicine you felt like…I feel like I can pay for these services if we are required to pay for them.” MAS006.*
**2.21**	*“Only if he is a professional. Because I am not certain that he is a professional. I heard some are not professionals; they just learned through work experience. But if he is a professional and knowledgeable, as a pharmacist, yes.” MAS012.*
**2.22**	*“I think I have good outcomes because whenever I check my blood pressure, it is really under control.” MAS014.*
**2.23**	*“Their presence helps to control sugar and high blood pressure. I remember vividly the good old days in this village; if you did not see a private doctor, you had to go to … [name of the hospital] or one or two chemists in town. You would sleep when you felt sick because it would be late. But nowadays, it is easy to seek services from nearby facilities.” MAS004.*
**2.24**	*“I would like them to be involved in raising awareness, providing education and actually advocating for people with diabetes, hypertension or these cardiovascular diseases so that people with such conditions know that they have the support of a pharmacist in their communities.” MAS005.*
**2.25**	*“I expect that they advise me on which community they have formed for people with diabetes who can have the same problems and exercise together. You know things if they are done together, I would suppose that these guys would do such things.*” *MAS009.*
**2.26**	*“I believe that the important thing is that people who work in pharmacies are pharmacists because they can help people in urgent matters; they can give first aid before patients see a doctor.” MAS012.*
**2.27**	*“Pharmacies should be everywhere, in the villages. With people who have been trained in the same way, who have the patience that when a person comes to explain himself, they listen to him and advise.” MAS006.*

#### Secondary role

##### Screening tests

Beyond prescription refills, respondents would go to the pharmacy to seek tests such as blood sugar tests when they feel sick or upon a community pharmacist's advice (Table 2, 2.06). Contrarily, most respondents do not take the tests at a community pharmacy because they 1) are unaware of such services at a pharmacy, 2) do the tests at the doctor's surgeries, or 3) possess testing devices at home (Table 2, 2.07 – 2.09).

##### Lifestyle counselling

Respondents indicated that community pharmacists encourage them to exercise regularly and watch over their diet. Community pharmacists monitor body weight by measuring the weight and height of the patients during prescription refills and advise accordingly (Table 2, 2.10 – 2.12).

##### Referral

Patients were probed on further action by community pharmacists in the case of abnormal readings. Respondents indicated that the pharmacist would advise them to see a doctor (Table 2.13).

##### Patient healthcare-seeking behaviour

Respondents routinely see a doctor every 3-6 months and visit a community pharmacist monthly for prescription refill. Both medical aid and out-of-pocket cash are used to pay a pharmacy bill. Despite their medical aid benefit, some respondents pay for their medication using out-of-pocket cash because the prices are affordable. On the other hand, some respondents buy certain medications (e.g. oral hypoglycemics) at a pharmacy and get others such as insulin, at the clinics due to higher insulin prices at the community pharmacies. Most respondents use certain pharmacies to get services. The reasons for using certain pharmacies were trust, affordability, reliability, convenience, and quality of service (Table 2, 2.14 – 2.19).

### Perceived community pharmacist care outcomes

#### Patient reaction

The respondents feel content and respected with community pharmacists' services. They appreciated community pharmacists for the reliable availability of medication, for involving patients in decision-making, and for quicker services (Table 2, 2.14, 2.16). When asked whether they would be willing to utilise and pay for extended community pharmacy services such as weight management programs, some respondents were willing to use and pay for such services while others would consider if the provider is qualified to provide such care (Table 2, 2.20 – 2.21).

#### Health outcomes

The respondents believed that community pharmacists positively impact their CVD outcomes. Community pharmacists have imparted knowledge of diseases, and their services encourage patients to seek healthcare services (Table 2, 2.22 – 2.23).

### Future expectations

#### Community pharmacist as a leader

Respondents believed that community pharmacists should increase their visibility by creating and leading communities and promoting health through disease education (Table 2, 2.24 – 2.25).

#### Standardisation of services

The respondents stated that community pharmacy should be regulated to meet specific training standards for standardising services across all pharmacies. (Table 2.26 – 2.27).

## Discussion

To the best of the authors' knowledge, this is the first study on CVD patients' perceptions of community pharmacists' services towards managing CVDs in Lesotho. The study adds knowledge to previous findings that have explored community pharmacists' and patients' perceptions of such role in CVD management. It was evident that CVD patients visit community pharmacies mainly for prescription refills. Most patients were not aware of other services besides prescription refilling. This implies that patients identify community pharmacists as ‘dispensers’ of medication, suggesting patients' lack of awareness of community pharmacists' scope of services and interest in utilising other services. Similar findings were echoed by Ogunbayo, Schafheutle^13^ and Almansour, Aloudah^14^ in the United Kingdom and Saudi Arabia, respectively^13^, ^14^.

On the other hand, some patients were aware of screening services but preferred to take the tests at the doctors' surgeries or use their own testing devices. This suggests community pharmacists' involvement in patients' self-care and presents variety for patients to choose from. Some patients reported receiving passive lifestyle counselling, rather than pharmacists being more proactive and supportive to ensure that patients get desired outcomes. Patients indicated that community pharmacists would take their weight measurements and advise them on diet modifications based on the results. The results imply variable patient experiences and utilisation of community pharmacy services, potentially due to a lack of awareness of such services.

Respondents routinely see a doctor every 3-6 months and visit a community pharmacist monthly for prescription refilling. The government of Lesotho recognises community pharmacies as an essential part of healthcare system that provides care and medicines^5^. However, community pharmacy practice is not integrated into Lesotho's primary healthcare structure. The established relationship model between community pharmacists, doctors and patients presents an opportunity collaboration between community pharmacists and doctors in Lesotho's fight against CVDs. Future research to focus on doctors' views on the role of community pharmacists in CVD management.

The location of pharmacies at the heart of communities makes them one of the most accessible and convenient healthcare services globally. The study established that the respondents look beyond accessibility and consider medication cost, trust and perceived quality of services. It is also evident that some patients only obtain services from specific pharmacies. They have selected pharmacies that they trust and are satisfied with their provision of services. The findings suggest a lack of standardisation in community pharmacists' services. Similar findings were previously reported in Nigeria, where patients showed dissatisfaction towards the quality of medication sold at some pharmacies and their preferences to use certain trusted pharmacies^15^. Pricing regulation should be considered to maximise patient satisfaction and confidence in utilising community pharmacists' care. Regulation of community pharmacies' pricing has been adopted and implemented in South Africa, Lesotho's immediate neighbour^16^. The enactment of the Lesotho Medicines and Medical Devices Control Authority Act 2023 in August provides for the establishment of the Lesotho Medicines and Medical Devices Authority, a body mandated with overall regulation of pharmacy practice^17^. The body presents a platform for improving pharmacy practice in Lesotho.

Respondents commended community pharmacists for being reliable through ensuring consistent medicine availability and responding to individual medical need. According to the Theory of Planned Behaviour, patients' expectations and perceptions of healthcare services guide their decision to utilise or not utilise them^18^. The theory further postulates that patients are more likely to utilise a healthcare service if they believe it is beneficial. In this study, patient satisfaction and trust were aligned mainly with medicine dispensing as the primary reason for the respondents to visiting a community pharmacy. For health promotion services, patients were willing to utilise and pay for extended community pharmacy services such as weight management programs provided that the provider is a qualified professional, underscoring a need for standardised community pharmacy practice and vigorous enforcement to build patient's confidence and trust. In the future, respondents aspire to see community pharmacists playing a more proactive leadership and supporting role by forming communities and raising awareness about CVDs. The respondent's willingness to participate in community initiatives is essential considering their lack of time and awareness of community pharmacists' services reported in previous studies^19^,^20^. Establishing communities could engage patients closely to ensure that their views are considered in decision-making for the success of healthcare programs. In Lesotho, patient groups exist such as the Lesotho Diabetes Association. The group brings together diabetic patients to provide disease education and support. Such platforms present an opportunity for community pharmacists to improve their visibility and impart knowledge of CVDs to the public.

The study had limitations. Firstly, because of the study's qualitative design, the use of a convenience sampling technique and a smaller sample size, the opinions might not represent those of the entire population of CVD patients. For example, the youths were not represented in the sample, and therefore, their opinions regarding community pharmacists might differ from the adult population. Secondly, the respondents CVDs such as heart attack and stroke were self-reported, subjecting data to information bias. Nonetheless, the authors are confident that the study provides views of CVD patients about their perceptions of community pharmacists in preventing and controlling CVDs and adds knowledge to the literature. The sample was represented by males and females from across two districts in Lesotho, both employed and unemployed, possessing secondary to tertiary education. The interviews were conducted in both the native language (Sesotho) and English to accommodate the respondent's language preferences. Therefore, the findings provide insights to community pharmacists and healthcare policymakers regarding the patients' perceptions of the role of community pharmacists in preventing and controlling CVDs.

## Conclusions

Community pharmacies are among the healthcare facilities most visited by CVD patients. However, it was evident that CVD patients visited community pharmacies primarily to refill prescriptions and were less aware of and underutilised health promotion services offered by community pharmacists. Nonetheless, their appreciation of community pharmacists' care and their willingness for continuous support highlight patients' satisfaction with the care they receive and present an opportunity for community pharmacists to raise awareness of their scope of work and expand their scope of care. There is a need to improve patients' confidence in community pharmacists through standardisation and enforcement of regulations to ensure that patients capitalise maximally in addition to community pharmacies being convenient. It is recommended that future studies should be conducted on a larger sample size of patients. The perspectives of other healthcare providers and policymakers regarding the role of community pharmacists in preventing and controlling CVDs should be explored.
